# How I Feel About My School—Adaptation and Validation of an Educational Well-Being Measure among Young Children in Sweden

**DOI:** 10.3390/ijerph18105075

**Published:** 2021-05-11

**Authors:** Rasmus Riad, Mara Westling Allodi, Eva Siljehag, Carina Wikman, Tamsin Ford, Sven Bölte

**Affiliations:** 1 Department of Special Education, Stockholm University, 11419 Stockholm, Sweden; mara.allodi@specped.su.se (M.W.A.); eva.siljehag@specped.su.se (E.S.); carina.wikman@specped.su.se (C.W.); 2 Department of Psychiatry, University of Cambridge, Cambridge CB2 1TN, UK; tjf52@medschl.cam.ac.uk; 3 Center of Neurodevelopmental Disorders (KIND), Centre for Psychiatry Research, Department of Women’s and Children’s Health, Karolinska Institutet & Stockholm Health Care Services, Region Stockholm, 11330 Stockholm, Sweden; sven.bolte@ki.se; 4 Child and Adolescent Psychiatry, Stockholm Health Care Services, Region Stockholm, 10239 Stockholm, Sweden; 5 Curtin Autism Research Group, Curtin School of Allied Health, Curtin University, Perth, WA 6102, Australia

**Keywords:** well-being, child self-report measures, young children, early childhood education

## Abstract

The well-being of children has received increasing attention in recent years. Nevertheless, we lack adequate brief self-report tools that enable us to consider young children’s well-being in policy evaluations and educational research. This study describes the adaptation and first validation of the Swedish version of How I Feel About My School (HIFAMS), a subjective well-being questionnaire suitable for children aged 4 to 12 years, which was originally developed in the United Kingdom (UK). Descriptive statistics with analysis of psychometric properties and confirmatory factor analysis (CFA) are based on the perceived well-being of 228 children in preschool and school aged 5 to 8 years old. The CFA endorsed a good fit to a one-factor model, and the scale showed moderate internal consistency (*r*_α_ = 0.63). The results are largely in line with the findings of the original HIFAMS. We conclude that the Swedish version can be applied in early preschool/school settings and could provide first-hand information about children’s well-being from the first years of education until elementary school grades. Practitioners in early education settings might benefit from HIFAMS assessments when seeking to understand children’s current well-being to provide support to children with special educational needs or children at risk for mental health issues. Researchers could use the HIFAMS to standardize child well-being evaluations in policy evaluations and interventional studies.

## 1. Introduction

Interest in the well-being of children has increased over the last several decades, and the adoption of the United Nations Convention on the Rights of the Child (United Nations, 1989) [1] has served as one turning point, specifically stating that children have rights in their own respect in addition to human rights [2,3]. The Convention on the Rights of the Child (UNCRC) emphasizes that children are not only individuals in need of additional protection but also that their voices should be heard in matters that concern them. In a broader sense, the UNCRC has recognized children as competent informers, indicating that their views and perspectives are to be considered and valued [3]. Along with its global ratification, the UNCRC has illuminated the need for measures and indicators to monitor the well-being of children and to evaluate adherence to children’s rights [2]. Historically, the measures of child well-being have mainly focused on objective indicators or have used proxies to assess child well-being [3,4,5]. Nevertheless, objective indicators may not be of relevance to the individual itself and the subjective and objective measures may even be at odds [6,7]. For instance, an adult may not be able to interpret that a child walking in circles is lonely, although at face-value seemingly occupied [8]. Relying on proxy reports from parents or teachers stems from the view of children as having limited cognitive or linguistic ability for self-assessment and that they are not capable of knowing or conceptualizing what is best for them [9,10]. However, despite being close to and caring for children, adults cannot know the child’s thoughts and feelings, and children might not disclose everything that happens in their lives [11,12]. In fact, children’s self-reported well-being and proxies rating well-being, have shown only a limited correlation [13,14,15]. Apart from this proxy problem, childcare providers and teachers might yield false positives, overidentify externalizing children as being at risk for mental health problems and to a greater degree recognize boys compared to girls [16,17,18]. To understand the state and well-being of children, there seems to be a need for multiple perspectives and effective screening tools, including from the viewpoint of children.

### 1.1. Well-Being

The concept of well-being can be understood as the presence of positive affect and an absence of negative emotions accompanied by perceived satisfaction with life [19]. However, well-being has a close connection to health, quality of life and happiness [9]. For instance, the Constitution of the World Health Organization [20] states, “Health is a state of complete physical, mental and social well-being and not merely the absence of disease or infirmity”. According to this definition, well-being is not only based on a certain moment but also comprised of several experiences and is something that lasts over time [9]. Well-being has also been described as the balance between current (personal) resources and challenges in life [21], contextualizing well-being as a dynamic concept dependent on the situation. Two major perspectives on well-being focuses either on happiness and pleasure (hedonic) or on the realization of one’s potential (eudaimonic) [9]. Within the hedonic tradition, the positive feelings are a goal in itself, whereas the eudaimonic perspective would consider positive well-being as a result or “byproduct” of a living in line with one’s personal views and values [22,23]. i.e., the eudaimoinc perspective focuses on the activity rather than the emotional outcome [23]. According to Waterman et al., subjective well-being translates to happiness and objective well-being to flourishing [23]. In summary, well-being is a multifaceted concept, including affective components, such as happiness, and it can differ due to context.

Drawing from literature reviews, the well-being of children has been operationalized as “happiness” in a number of studies [24,25]. The positive affect of happiness is one of the first emotions recognized by children and can be distinguished by children from the age of 5 [26,27]. Happiness can also have connections to other abilities in life, including prosocial behavior, creativity and problem solving and coping behaviors [28,29]. Nevertheless, the combination of measuring both positive and negative emotions has proved useful to identifying youths needing support [30,31].

Some existing measures have been developed to gauge well-being in educational settings, such as Multidimensional Students’ Life Satisfaction [32] with a subscale devoted to school-related questions, and the Personal Well-being Index: School Children, has been further developed to include five items about school [33]. Monitoring well-being in the school context has been suggested to improve inclusion and enable sustained support to vulnerable children at risk for school failure [34]. Indeed, the educational setting is a central arena for intervention in which children can be reached and supported. Hascher [35] describes that happiness in school might not by itself foster well-being but produce a positive bias for learning in school. This description further denotes happiness as a purposeful dimension in the learning environment that contributes to attainment and development. Nevertheless, none of the aforementioned measures target children younger than 7 years old, which excludes a great number of children, considering that 90% of children in Organisation for Economic Co-operation and Development (OECD) countries are enrolled in early childhood education or care from the age of 5 [36]. Several authors [10,37,38,39] have previously identified the lack of instruments of subjective well-being focusing on younger children. The limited reliability for subjective measures for younger children has been suggested to be one of the reasons for the scarce number of instruments [38,40]. In terms of validity, response patterns for younger children have been observed to be more extreme compared to older children [41] and potentially influenced by strategies [42], such as trying to provide “correct” answers [43,44].

However, subjective measures have successfully been used with younger children, such as the Pediatric Quality of Life Inventory [45] and the Group Climate Instrument [46]. Questionnaires might require adaption to suit younger children. For instance, the barriers of literacy and cognitive development can be addressed using emoticons, which have been suggested to enhance children’s understanding of questionnaires [47,48,49]. In addition, it might be appropriate to lessen the number of response options since fewer options lessen the load on verbal memory [50,51,52]. These proposed modifications might improve the reliability of questionnaires targeting young children [50].

### 1.2. Well-Being of Young Children in Sweden

The well-being of young children has recently received national attention in Sweden. In 2019, the Ombudsman for Children in Sweden [53] acknowledged the limited information regarding the perception of preschool from children’s perspectives. Subsequently, an online questionnaire was sent to children at 5 years of age (*n* = 219). The majority (92%) of the children responded both that they were pleased with how they were greeted upon arrival at preschool and that they had positive feelings when they left. However, one quarter of the children expressed negative feelings upon arrival at preschool [53]. The applied questionnaire provided new insights into the perspectives of the subjective well-being of younger children. However, the Ombudsman for Children has not yet announced any further assessment or follow-up. In addition to these insights about the current well-being of young children in Sweden, new statutes have been introduced. In January 2020, the UNCRC [1] was adopted into Swedish legislation. The implementation of UNCRC is intended to improve adherence to the Convention, which has been lacking at both the municipal and state levels [54]. To improve compliance with the UNCRC and provide children with a voice in matters that concern them, additional measures are needed. A validated measure within the educational context is convenient for evaluating both policy and provisions, as well as mediating special educational interventions.

### 1.3. How I Feel About My School

How I Feel About My School (HIFAMS) is a questionnaire developed by Ford [55] that is suitable for children from 4 years old up to the age of 12 years old. The questionnaire originated from a project aiming to support children and teachers in school [56]. Since it spans the early years of education through the middle school years, it is a promising indicator that has been included in longitudinal studies of educational quality and mental health from early ages [57]. The HIFAMS is the result of collaborative work involving several professions and parents with the aim of optimizing the content and procedure [13]. The questionnaire addresses seven questions of perceived well-being in the educational setting, including satisfaction with teachers, peers, classrooms, playgrounds, the transition to school, work and school in general. The HIFAMS could provide valuable insight into the subjective well-being of young students, a population in which the current knowledge is limited.

The validation study of the HIFAMS [13] reported moderate two-week test-retest reliability [Spearman’s correlation (*r*_s_) = 0.62; 95% CI: 0.54 to 0.69] and moderate internal consistency (Cronbach’s alpha *r*_α_ = 0.62). Furthermore, HIFAMS demonstrated a slightly higher mean score for girls of 0.37 points [13], based on a sample of 2345 children aged 4 to 8 years old. A total of 4.9% of the variability was accounted for by school differences, and the remaining 95.1% was due to differences between pupils within schools [13]. On the HIFAMS, children at risk of exclusion from school have reported themselves to be less happy than an unselected community sample (mean difference = 2.2; 95% CI = [1.5, 3.0]; *p* < 0.001) [13]. Since the children at risk were expected to have a lower degree of happiness in school, the HIFAMS might be able to distinguish relative differences between groups. Moreover, the validation study presented a confirmatory factor analysis (CFA), indicating a one-factor model with factor loadings ranging from 0.48 to 0.74 for six of the seven items. The item regarding playground provided factor loading ranging from 0.13 to 0.26 [13].

Apart from validation [13], the HIFAMS has previously been used to evaluate the support of siblings for children with special educational needs or disabilities [58]. Siblings reported slightly increased perceived happiness after the intervention, although the difference was not statistically significant [58]. Furthermore, the HIFAMS has been used to compare relative differences for children born at different time points during the year [59]. Children with suspected Attention deficit hyperactivity disorder (ADHD) (*n* = 47) have also been reported to have significantly lower HIFAMS scores than their peers (mean difference −1.2, 95% CI = [−0.5, −1.8]; *p* = 0.001) [57]. The mean score of the HIFAMS at the group level was approximately 11 out of 14 [13,58]. HIFAMS has also been used as a bridge to introduce children to discuss and reflect upon feelings and emotions [60].

The present study describes the translation and adaption of the HIFAMS subjective well-being measure [13] and its first validation with young Swedish preschool and school students. The first aim is to report the psychometric properties of the well-being construct measured with the HIFAMS in this sample, specifically the internal consistency, interitem correlation and measurement model obtained with confirmatory factor analysis. The second aim is to examine the answers with respect to gender and age group.

## 2. Materials and Methods

### 2.1. Instrument

The HIFAMS questionnaire is currently available in English, Italian and Persian. The approved Swedish version will be accessible at the website, along the other versions [61]. The scale consists of seven questions scored as Sad (0 points), OK (1 point) and Happy (2 points) with a total range of 0–14 points. A higher total score of HIFAMS indicates a higher degree of perceived happiness. For each question, children assess their happiness or sadness on a single page of paper by marking their responses alternatively through verbal responses or through pointing for younger children. The seven questions include how children feel on their way to school or preschool, in the classroom, during work or an activity, on the playground and while thinking of the other children, as well as about the teacher and the school as a whole.

### 2.2. Translation and Adaption

Proficient experts translated the original version of HIFAMS into Swedish. Some adaptions were required for the preschool version due to organizational and structural differences between the education systems in the UK and Sweden. In Sweden, children at the age of 5 years old attend preschool settings that are not organized as one classroom but are deployed in various rooms; the activities are not called “work” but are to a large extent informal and play-based activities. The preschool version adaption concerned the wording of “classroom”, which was substituted with “my section of preschool”. Furthermore, “doing my work” was substituted with “activity”. These adaptations were discussed with and approved by the author Tamsin Ford in 2019. The two questionnaires (preschool and school versions) were subsequently back-translated and compared to the original version, and the final versions were approved by the author. The final preschool version was piloted with preschool children before the start of the study, leading to additional instructions regarding the wording of “activities”. Assessors were instructed to provide examples of activities, such as painting, drawing or building with blocks. The provisional script from the original version was also translated and adapted for Swedish conditions.

### 2.3. Participants

The participants were attending preschool and elementary school. The preschool sample (*n* = 85) was enrolled in an early literacy intervention, and the school sample (*n* = 143) was enrolled in a socioemotional climate intervention. The mean age of the preschool children was 5.47 years (*SD* = 0.25) and 8.4 years (*SD* = 0.4) in the school-aged children. The preschool sample attended 10 preschools located in districts with different socioeconomic characteristics (large city, medium sized city, commuting municipality), and the school sample attended four schools located in 4 districts with different socioeconomic characteristics (large city, commuting municipality), according to the official classification of municipalities [62]. The demographic data are presented in Table 1.

### 2.4. Procedures

#### 2.4.1. Preschool Assessment

Trained research assistants (*n* = 9) followed the provisional script of the HIFAMS and ascertained that the children understood what was expected of them during the assessment. The provisional script included information that there were no right or wrong answers, and the purpose was to understand the child’s feelings about the preschool environment. In addition, children were prepared with sample questions, ensuring they had a proper understanding of the scale beforehand. If a child did not provide a reasonable answer, more sample questions were presented to the child, until the child showed a satisfactory level of understanding. During testing, children were informed about the voluntary nature of the testing and that they could leave at any time. The preschool children also had the possibility of bringing staff to the assessment as support. Children were offered the opportunity to answer the HIFAMS questionnaire in a separate room at the preschool with the possibility of opting out. The nonverbal response of pointing was offered during the whole session through visual support, displaying the emoticons. The research assistant confirmed responses by saying them aloud. The last provided response by the child was considered to be final. Nine trained research assistants, including the first author, collected responses during December 2019.

#### 2.4.2. School Assessment

The fourth author (C.W.) collected the school sample data in class, following the prescribed instructions for children of this age. The researcher collected the data in a group setting and read the questions aloud, yet the students completed the questionnaires individually, with guided support if needed. Additional concern was given to the possibility of screening out answers due to potentially sensitive questions concerning both teachers and classmates. Children completed the HIFAMS individually with guided support, if needed. Data for school samples were collected during November 2019.

### 2.5. Analysis

Data analysis was performed using SPSS (version 26) (IBM, Armonk, NY, USA) and Mplus (Muthén & Muthén, Los Angeles, CA, USA) [63] software. Participant characteristics and descriptive statistics of the HIFMAS were calculated using means, standard deviations, ranges and frequencies. The total HIFAMS score was summarized according to previous studies (Sad = 0; OK = 1 and Happy = 2). The reliability of the HIFAMS was assessed by calculating Cronbach’s alpha. Item-total correlations were computed for each of the seven items using nonparametric testing due to the categorical structure of the HIFAMS; the data were not assumed to be normally distributed.

To assess construct validity, confirmatory factor analysis (CFA) was conducted to test the one-factor measurement model of the 7-item HIFAMS, similar to a previous validation study [13]. The CFA was assessed for fit based on several fit indices, the comparative fit index (CFI), root mean square error of approximation (RMSEA) and standardized root mean square residual (SRMR). Exact fit was expected with a nonsignificant chi-square (*p* > 0.05). Approximate fit was decided by a cutoff value ≥0.08 for SRMR and standardized residuals < 0.10, according to residual interpretations by Kline [64] and for RMSEA with an estimated value less than 0.05 and an upper limit 90% confidence interval less than 0.08. Acceptable fit for CFI was decided based on a value >0.95 [65]. Modification indices could be analyzed to understand model fit and whether any indicator affected the model fit [66].

A multiple indicators multiple causes model (MIMIC) is a particular form of structural equation modeling that contains two parts: a structural model that specifies the causal relationships among latent variables; and a measurement model that expresses the relationships between a latent variable and its indicators [64]. The MIMIC model includes additional dummy variables that are believed to influence the latent construct imposed on the factor model [66]. A MIMIC model was used to regress the concept of well-being on the dummy variables of age group (preschool = 0 and school = 1) and gender (male = 0 and female = 1). To assess model fit, the weighted least square mean and variance adjusted (WLSMV) estimator in Mplus software were used. The WLSMV does not assume normally distributed variables and is recommended for categorical data [66].

### 2.6. Missing Values

The data set contains 32 (14%) instances with missing data, which were item nonresponses or coding errors, i.e., responses placed between emoticons (*n* = 21) instead of on them or skipped question (*n* = 2). The remaining missing data were all absent at data collection and had missing values for all of the variables (*n* = 9). Visual inspection of the missing value pattern indicated it to be random. To remedy the missing data, multiple imputations were run on the data set using a fully conditional specification, and the imputed sample consisted of 219 cases, of which only the entries with completely missing data were excluded from analysis.

## 3. Results

### 3.1. Descriptive Statistics

Most of the children reported relatively high levels of happiness within the preschool and school. Item #6, regarding the teacher, had the greatest percentage of children responding they were happy (72.6%), and item #1, describing their feeling on their way to school, had the smallest percentage of children reporting that they were happy (56.2%). The next highest percentage was on the item concerning thinking about being on the playground (70.6% were happy). The highest percentage of children reported being sad when thinking about school (10.7%) and when doing schoolwork or activities (8.3%). The other response percentages to the respective items can be found in Table 2.

The preschool sample had a response rate of 90.6% (*n* = 77) and resulted in a mean total score of 10.20 (*SD* = 2.45) out of the maximum of 14. The girls in the preschool sample had a lower mean score (10.0) than the boys (10.5). The school sample had a response rate of 99.3% (*n* = 142), with a mean score of 11.16 (*SD* = 2.27). In the school sample, girls scored themselves as higher than boys, 11.5 and 10.8 points, respectively. A qualitative analysis of the total distribution indicates a ceiling effect, with 15% scoring “happy” on all items—a pattern not observed for the other responses, i.e., only responding “OK” or “Sad”. The total sample consisted of 219 children, of whom 57% were girls. The total mean score was 10.82 (*SD* = 2.6).

### 3.2. Item Values and Item Totals

The item with the lowest scores was item #1, with a mean of 1.48. The items with the highest overall scores were items #4 and #6 regarding playground/outside and teachers, with means of 1.64 and 1.65, respectively. The item with the highest correlation with the total score was the general question “when I think about my preschool/school”, with a correlation factor of 0.53 (Kendall’s tau b correlation, *p* < 0.0001). The item with the lowest correlation with the total score was the question about playgrounds/outdoors, “when I think about the playground” (0.39, *p* < 0.0001). Item #4 did not generate significant correlations (*p* > 0.05) with four of the other items; however, all of the items had a significant (*p <* 0.05) correlation with the total score. The total sample mean score was 10.82 out of a maximum of 14.

### 3.3. Internal Consistency

The total sample demonstrated moderate internal consistency (Cronbach’s alpha value of 0.63), and no improvement in reliability was obtained by deleting any of the items.

### 3.4. Construct Validity

**Figure A1:**
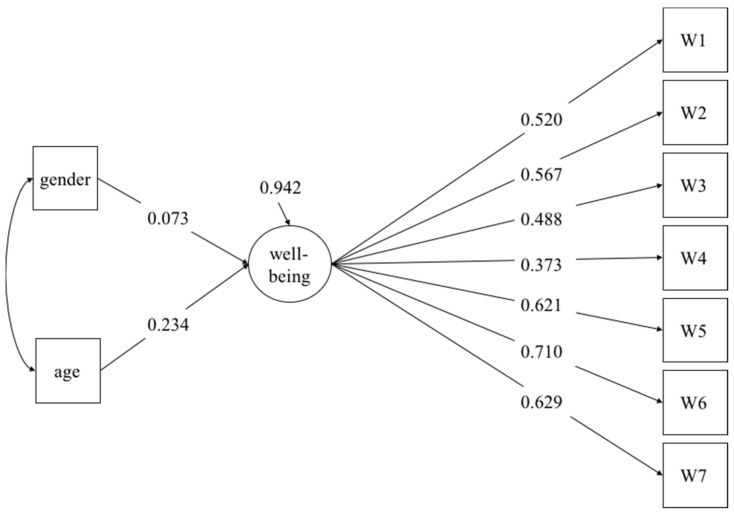
Path-model of HIFAMS, Swedish version, with standardized estimates.

The CFA indicates a one-factor model of well-being with an overall good fit to the data (χ^2^ = 16.572; *df* = 14; *p* = 0.280; RMSEA = 0.29; CI = 0.000−0.074; CFI = 0.99; SRMR = 0.049) (See Table 3). All of the standardized residuals of correlations were relatively small (<0.10). Standardized factor loadings ranged from 0.38 to 0.70 (factor loadings available in Table 4). Subsequently, gender and school setting were added to the model using the MIMIC approach [66]. The results demonstrate that girl had a non-significantly higher mean score (*β* = 0.076; S.E. = 0.089; *p* = 0.393), and older students (mean age = 8.4) had a significantly higher mean score (*β* = 0.256; S.E. = 0.101; *p* = 0.011) on the HIFAMS than younger students (mean age = 5.5) (see Figure A1 in Appendix A). The *y*-standardized estimate coefficient can be interpreted as Cohen’s *d* [66], indicating the standardized score difference between specified groups. With respect to gender, girls scored higher on standardized points (*d* = 0.145; *p* = 0.388) than boys, whereas the total score for older students was higher than that for younger students in preschools in this sample (*d* = 0.488; *p* = 0.007). Adding the dummy variables did not drastically affect the model fit, indicating a good approximation of the model in comparison to the data (see Table 3). The path-model, including dummy variables, can be found in Appendix A Figure A1.

## 4. Discussion

The subjective well-being of children has received increased attention since the introduction and ratification of the Convention on Rights of the Child, not the least when incorporated into national legislation. The lack of self-report instruments targeting the younger population is a gap in the literature and practice. This gap calls for effective tools to enable the evaluation of current practices. In addition, such an instrument could be used as an indicator or outcome in longitudinal studies and interventions. Therefore, the present study aimed to validate and analyze some psychometric properties of a translated and adapted version of the HIFAMS for preschool and school children aged 5–8 years old in Sweden. We expected the Swedish versions of the HIFAMS to indicate the same one-factor structure, as well as similar internal consistency as previous validations [13].

The CFA showed that a one-factor model had a good fit to the data. The findings showed moderate and satisfactory internal consistency for the HIFAMS, similar to previous reliability testing [13]. We found that, among the seven items, item #4 regarding the playground had the lowest factor loading (0.38). Item #4 also displayed limited correlation with the other items. As explained by Allen et al. [13], the playground offers a nonteacher organized environment where bullying can occur. The playground is synonymous for schoolchildren with breaks from schoolwork, and within preschools, it offers a varying amount of space and activities. The limited intercorrelation for item #4 might be explained by children perceiving this situation differently than other aspects of school. In a previous systematic review of mental health and well-being in a Swedish school [67], some children referred to breaks and being outside as the only positive occasions during the day. At the same time, some children expressed that breaks were stressful and lonely, leaving them rather exposed [67]. To obtain a better understanding of different aspects of well-being at school a latent profile analysis might identify potential well-being subgroups within the data e.g., Virtanen et al. [68]. The outdoor milieu is, however, an important part of the school environment and thus is meaningful to include in the construct of well-being at school.

The gender differences were not significant in this study, with a nonsignificant 0.08 higher mean score for girls overall. In comparison, the UK sample presented a significantly higher mean score for girls of 0.39 [13]. The lack of a significant gender difference could be attributed to the small sample size within this study and could be investigated further in a representative larger sample. However, according to a Norwegian study of self-reported well-being in school (*n* = 268, grade 1–10), no gender differences were found in that context either [69].

The age factor relating to preschool and school in this study showed a positive relationship with the HIFAMS. Children aged 8 in the school setting scored themselves as happier than children aged 5 in the preschool (*d* = 0.488; *p* = 0.007). The age variable has previously not been reported as a significant predictor of the total score [13,59].

The mean total score, both for subgroups and the total (10.85), was within the expected range, referring to an earlier study in which the majority had mean scores close to 11 and 2.4 standard deviations [13]. The cross-sectional design and the small sample did not allow us to draw conclusions about the results indicating age differences regarding self-reported well-being in our context. This issue could be investigated with a longitudinal approach, following children over time from preschool to school settings.

### 4.1. Limitations

The main limitation of this study is the lack of an additional concurrent or convergent measure to validate the HIFAMS. A second measure could also improve the understanding of the observed ceiling effect and the representativeness of these responses. For instance, students’ self-reports can be affected by motivation. Novelty and disruption effects imply that students might respond unusually well to an innovation (in this case, answering a new type of questionnaire, with another adult rather than usual staff) that disrupts the routine, which can be a threat to validity. This effect might have especially occurred in the school setting, since the students’ experiences of the innovation were quite positive. Although most children provided valid responses, some school children marked their answers in between the emotions (scores). This issue should be addressed in the form by clarifying with pictorial aid how exactly a response should be provided. During the piloting, the preschool children were encouraged to elaborate their answers. For instance, one child clarified the feeling “sad” on the way to preschool due to reluctant feelings of being separated from his mother. This response indicated a contextualized and relevant response to the question. When a child in preschool did not provide a reasonable answer to the first few sample questions, additional questions were offered along with refined instructions. Nevertheless, a qualitative analysis might be needed in order to ensure that all children have a proper understanding of the scale and questions such as Montserrat et al. [52]. Moreover, the answers that children provided might in some cases have been influenced by social desirability motives rather than mirroring their genuine feelings [43,44]. However, children in Swedish preschool are somewhat accustomed to being asked upon their opinion in school settings and make decisions [70,71]. In addition, all children were explicitly told that the questions in HIFAMS were based on our genuine interest in their feelings and that they would not be judged based on their replies. Although our results are within the same range as previous psychometric studies of the HIFAMS, subjective well-being is contextual and should be considered in relation to its own cultural context [9]. The relationship with socioeconomic status (SES) was reported in previous studies from the UK but was not assessed in this study since individual data on SES are not available in our context, if not collected specifically. SES showed a significant relationship with children’s well-being in the UK studies, with children at-risk showing lower scores, and this relationship could be investigated in a future study.

### 4.2. Implications

In the report from the Ombudsman for Children in Sweden [53], the large majority (92%) of included preschool children (*n* = 219) responded that they felt comfortable or at ease in preschool. The results from this study and distribution in percentages (Table 2) are in a sense similar: even the present results indicate that a minority of children were sad. However, on the HIFAMS, the responses are anchored in Happy, OK and Sad, which are less cognitively challenging compared to multiple responses and different response alternatives for each question [52]. The HIFAMS is nuanced and provides richer information compared to a simpler type of questionnaire with only two alternatives. In fact, in this study, on the seven items, between 20% and 40% of the answers yielded the middle choice (OK), suggesting that the children’s perceptions of these topics could be improved. The children who answered that they were happy ranged between 56% and 72% on the seven HIFAMS items, thus a more modest percentage compared to the study from BO. With answers on seven items graded on a 3-point Likert scale, we assume that it would be possible to detect changes over time, for instance, after policy improvement. The translation and adaption of certain items for younger children (classroom/section; work/activity) might be useful even for other educational contexts with a similar system and structure. For instance, within the Nordic countries, formal education does not begin until age 6 and is less formalized [72,73]. Moreover, HIFAMS can be used as first tier of assessment that leads to additional discussion with the child [60].

Since its psychometric properties seem satisfactory even in our context, while the instrument is theoretically sound, developmentally adequate and easy to use, we believe that the HIFAMS could be employed in evaluations performed by service providers and national agencies in Sweden.

## 5. Conclusions

As suggested by previous research, happiness and well-being in school can have implications for several outcomes later in life [28,29], and well-being can be understood as a positive bias for learning [35]. The adoption of the Convention on the Rights of the Child is a shift toward empowering children’s perspectives. The HIFAMS can, in a straightforward manner, capture self-reported well-being for young schoolchildren in Sweden and provide valuable knowledge to practitioners and stakeholders. Recognizing children’s points of view could further affect, improve and influence preschool and school practice.

## Figures and Tables

**Table 1:** Sample characteristics of age, gender and school setting.

Setting	Number of Participants (% Female)	Schools	Classes	Age, Mean (*SD*)
Preschool	85 (57)	9	10	5.5 (0.25)
School	143 (51.7)	4	8	8.4 (0.4)
Total	228 (53.9)	13	18	7.4 (1.47)

**Table 2:** Item responses by item on the Swedish version of the HIFAMS.

Item Number and Question How I Feel…	Sad %	OK %	Happy %
#1. On my way to school	3.3	40.5	56.2
#2 When I am in the classroom/unit	6.6	34.4	59.0
#3. When I do an activity/schoolwork	8.3	32.4	59.3
#4. When I am in the playground	5.6	23.8	70.6
#5. When I think about the other children	6.2	37.4	56.4
#6. When I think about my teacher	7.4	19.9	72.7
#7. When I think about school	10.7	28.0	61.2

Note: HIFAMS = How I Feel About My School.

**Table 3:** Confirmatory factor analysis of the HIFAMS by WLSMV estimator with fit indices for 3 models.

Model	χ^2^	*df*	CFI	SRMR	RMSEA	90% CI RMSEA
One-factor model	16.572 (*p* = 0.280)	14	0.988	0.049	0.029	0.000–0.074
+gender	20.004 (*p* = 0.458)	20	1.0	0.047	0.001	0.000−0.058
+gender +age	29.701 (*p* = 0.280)	26	0.983	0.053	0.025	0.000−0.061

Note: Gender = female/male; age = 8.4/5.5; CFI = comparative fit index; SRMR = standardized root mean square residual; RMSEA = root mean square error of approximation.

**Table 4:** Standardized estimates, one-factor model and standardized estimates.

Well-Being One Factor	Standardized Estimates	S.E.	Est S.E.	*p*-Value
#1	0.53	0.081	6.562	<0.001
#2	0.55	0.076	7.303	<0.001
#3	0.49	0.075	6.539	<0.001
#4	0.38	0.084	4.557	<0.001
#5	0.61	0.070	8.822	<0.001
#6	0.70	0.067	10.506	<0.001
#7	0.63	0.063	9.942	<0.001

Note: Gender = female/male; age = 8.4/5.5; CFI = comparative fit index; SRMR = standardized root mean square residual; RMSEA = root mean square error of approximation.

## Data Availability

The data presented in this study are available on request from the corresponding author.
